# Dr. Frederick Preiss

**Published:** 1906-07

**Authors:** 


Dr. Frederick Preiss, of Buffalo, one of our prominent physicians, a graduate of Trinity Medical College, Toronto, 1890, died at the Sisters' of Charity Hospital, May 30, 1906, aged 37 years. He underwent a severe surgical operation about a fortnight before his death, from which he rallied promptly and it was expected that he would recover. Dr. Preiss had been in active practice in this city since his graduation and a few years ago was joined by his brother, Dr. William Preiss, who was associated with him until his death. Dr. Preiss was a member of the Medical Society of the County of Erie, of the Buffalo Academy of Medicine, and of the Medical Union. Funeral services were conducted in this city, June 1, at which the honorary bearers were Dr. George L. Brown, Dr. A. H. Briggs, Dr. R. L. Banta, Dr. William C. Phelps, Dr. Stephen Y. Howell and Henry Zipp. The remains next day were taken to Middleport, Ont., Canada, for burial.

